# Culture, heritage looting, and tourism: A text mining review approach

**DOI:** 10.3389/fpsyg.2022.944250

**Published:** 2022-08-08

**Authors:** Sandra Maria Correia Loureiro, Amr Al-Ansi, Hyungseo Bobby Ryu, Antonio Ariza-Montes, Heesup Han

**Affiliations:** ^1^ISCTE-Instituto Universitário de Lisboa and Business Research Unit, Lisbon, Portugal; ^2^Faculty of Hospitality and Tourism Management, Macau University of Science and Technology, Taipa, Macao SAR, China; ^3^Food Franchise Department, College of Health Sciences, Kyungnam University, Changwon-si, South Korea; ^4^Social Matters Research Group, Universidad Loyola Andalucía, Córdoba, Spain; ^5^College of Hospitality and Tourism Management, Sejong University, Seoul, South Korea

**Keywords:** cultural heritage looting, heritage destruction, public access, human rights, cultural heritage preservation, protection of cultural property

## Abstract

Tourism scholars have been devoted to exploring the significance of cultural heritage in generating economic, environmental, and social values. However, limited efforts were found to verify potential threats that demolish these values such as looting issue in the global heritage tourism industry. Therefore, this study has reviewed extant publications to demonstrate the potential emerged textual clusters discussed by previous studies. It also summarized the network distribution of articles journals and authors’ affiliations to capture the mobility and diversity with a focus on the business and tourism management field. Hence, the core clusters discovered were related to heritage destruction, public access, world heritage, human rights, cultural heritage preservation, and protection of cultural heritage in the event. The results have established theoretical insights and research agendas for future tourism studies, while it determined critical drawbacks in employing technology tools including virtual reality, augmented reality, and artificial intelligence for cultural heritage preservation/protection.

## Introduction

Cultural heritage is the core identity and the national character of communities across the globe (Giakoumis, 2020; Trinh et al., 2020). Losing its authenticity and historical values leads to demolishing the community principles (Brodie and Renfrew, 2005; Al-Ansi et al., 2021; Saifi, 2021). Scholars from different fields attempted to demystify the invisible impacts involved along with the evolution of cultural heritage looting. From the tourism management perspective, it tends to be an essential part of a global organized crime that created an active illegal market (Bowman, 2008; Campbell, 2013; Greenland et al., 2019). This global issue has established a barrier for many governments, local authorities, and international organizations to restrain its rapid growth due to its striking trades of looted antiquities. The global effort regarding this issue has called for several emergency initiatives to overcome and eliminate its uncontrolled growth to protect the nations’ valuable possessions (UNESCO, 2016). The global illegal business of cultural heritage has far-reaching consequences on the coherence of communities, sustainable principles, tourism management, transparency values, and human rights (Mackenzie and Yates, 2016). Thereby unraveling the intricacies of this black-market nexus with the social development of communities and characteristics of human values is a critical matter.

Cultural heritage looting is defined as an illegal act breaking the global business law in dealing or trading with cultural heritage objects such as antiques, artifacts, or any historical items (Al-Ansi et al., 2021). In a sense, it is committing a crime against the cultural heritage and human civilization values that were inherited from past generations. Many global organizations (e.g., governmental and non-governmental) have apprehended it as a total threat to society, the economy, and the environment. The United Nations Educational, Scientific and Cultural Organization UNESCO have acknowledged the drawbacks among global state members to cooperate in fighting closely against this black market (UNESCO, 2016). Recently, the International Criminal Police Organization INTERPOL has urged the global states to combat this phenomenon through enhancing collaborations and partnerships. The absence of an effective tool and plan to combat cultural heritage looting has raised the illegal trade activities of antiques and artifacts across the world during past decades (INTERPOL, 2019).

As a shift from commitment into an action, an early alarm was reported by the international council of museums ICOM to protect many valuable cultural heritage objects inherited from the most vulnerable areas across the world including [Asia: Afghanistan, Cambodia, China; Africa: Nigeria, Mali, Ghana, Chad, Senegal, Cameron, Burkina Faso; South America: Mexico, Colombia, Brazil, Chile, Peru, Ecuador; and the Middle East: Egypt, Yemen, Iraq, Syria, Libya] (ICOM, 2020). The proliferation of looted cultural heritage property has been observed in many art houses and auction centers across Europe, United Kingdom, and United States (Altaweel, 2019).

In turn, the increased action of looting cultural treasures reflects on the local society characteristics and harms values including identity and authenticity (Al-Ansi et al., 2021). Past literatures have overviewed the critical role of preserving and protecting cultural heritage to reinforce new tourism development and sustainability (Trinh et al., 2020). However, the efforts conducted by previous scholars have highlighted limited insights and perspectives about the global phenomenon of cultural heritage looting which produced an insufficient understanding of its economic, environmental, and social repercussions.

The twofold scopes of this global crime and phenomenon in looting practice involved theoretical and practical dimensions that spawned a complex topic to understand its processes, aspects, and attributes through the past years. Even though some scholars’ endeavors have addressed its critical entangled and impacts from different perspectives, the intricate spheres of this global dilemma require more sophisticated work that explores the present paths of this illegal active market. To delve into this topic, academia and other relevant educational fields must decipher the unseen zones of this phenomenon. Thereby, academics, heritage managers, and non/governmental agencies are required to reshape their present strategies when dealing with this global dilemma through assessing their goals and reviewing previous studies’ efforts in protecting the cultural heritage sites (Mualam and Alterman, 2020). This can help to create a plausible approach for future studies and its theoretical orientations. It also can demonstrate an implemented guidance to foster management process skills to curb its illegal business market. Therefore, cultural heritage and looting is a relevant topic for tourism in cultural sites that deserve further attention. Yet, so far, no past research provides a perspective on the extant literature on the topic. Thus, this report seeks to answer the following research question: “What tourism research has been conducted on cultural heritage and looting and how future research could evolve from now?”

Therefore, this study aims to provide the main clusters that emerge from the prior studies and suggestions for future research on the topic. It also attempts to give an initial light to pave a visible path on the topic by assessing and reviewing the prior published documents after a comprehensive search and collection of them, presenting the main clusters and highlighting the future research agenda.

Our study contributes to the tourism management in three ways. First, it is the first comprehensive review of cultural heritage and looting research, covering 30 years of publications on the topic with 30 scientific articles and 16 other documents. Second, we point out the scientific journals where this topic has been published, the network of authors and the countries where their universities are located and the cluster analyze with six core clusters, namely: heritage destruction, public access, world heritage, human rights, cultural heritage preservation, and protection of cultural property in the event. Finally, we outline the future research agenda.

## Materials and methods

**FIGURE 1:**
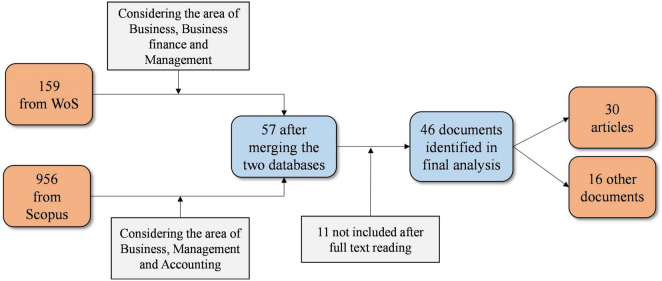
Documents search and selection.

We collected documents on cultural heritage and looting from two well-known online libraries—Web of Science (WOS) and Scopus—using the following query applied to the title, abstract, and keywords: (“Culture* heritage” AND looting). Figure 1 illustrates the documents found in both databases. When filtered for business, economic and finance/accounting and merging, 57 documents remain. Other areas are associated with agriculture, biology, environmental science, physics, computer science, earth and planetary science, or art and humanities and are not considered due to the focus on business and tourism management. The full text reading was performed by two researchers independently regarding the consistency standards suggested by Macpherson and Holt (2007). This process led to an agreement of excluding eleven documents, with a Cohen’s Kappa coefficient of >0.85 (Cohen, 1960).

The VOSViewer software tool was then used to conduct the network analysis (Van Eck and Waltman, 2010). The network analysis performed includes journals and researchers and is built on co-authorship. VOSViewer employs visualization-of-similarities (VOS) mapping to create two-dimensional bibliographic networks (Waltman et al., 2010). The weakly (strongly) related nodes emerge far from one another (close together) (Van Eck and Waltman, 2014). Co-authorship analysis explores the social relationships among authors and their country of affiliations and equivalent impacts on the development (Acedo et al., 2006) of the cultural heritage and looting research.

Co-authorship is relevant because allow to understand how authors interact among themselves and what are the countries of affiliated institutions (Acedo et al., 2006; Cisneros et al., 2018). The increase theoretical and methodological complexity of the research leads authors to collaborate among them and this analysis allow to understand the network and who are the most prolific authors (Tahamtan et al., 2016). The insights that come from co-authorship analysis can be used to stimulate new research and collaborations. Therefore, the information about authors affiliation that come from both WOS and Scopus is introduced in the VOSViewer software to be able to trace the networks.

We also used MeaningCloud text mining tool for text clustering. The MeaningCloud tool analyze the text of the papers and create clusters, each one representing text that is similar (Spinakis and Chatzimakri, 2005); groups (clusters) by analyzing the text of each article (Fan et al., 2006).

MeaningCloud software uses Text Clustering API that allows to uncover the implicit structure and the meaningful subjects embedded in the contents of the articles. This API takes a set of texts and distributes them in groups (clusters) according to the similarity between the contents of each article. The aim is to include in each cluster articles that are very similar to each other and—at the same time—highly different from the ones included in other clusters.

The clustering process (1) employs lemmatization technology to consider all the morphological variants of a term (e.g., high/higher/highest), (2) allows to define words that should not be considered in the analysis process due to their little semantic relevance, (3) groups the articles according to their relevance with respect to the context in the analyze and not purely textual similarity, (4) assigns to each cluster a name which semantically represents its contents (Fan et al., 2006; MeaningCloud, 2022).

## Overview of the documents

The group of the other documents (16) is composed by three books, eleven book chapters, one review, and one editorial. The editorial refers to the introduction of the Timothy’s (2017) book, which deals with issues of conservation, interpretation, impacts of tourism and the management of those impacts. The review is a perspective on the interactions and expectations of community members, archeologists, and the state as they interact within the archaeoscape of Uxbenká (Parks, 2010). The three books address issues relating to law and restitution (Stamatoudi, 2011), or is devoted to antiquities are the cultural property (Cuno, 2012), or even deals with the public health humanitarian responses to natural disasters Chan (2017). As for book chapters, the majority belongs to the book edited by Chappell and Hufnagel (2014) dedicated to art and antiquity crime. Other chapters are more focused on heritage, museums, and galleries (e.g., Corsane, 2005; Campelo et al., 2018).

**FIGURE 2:**
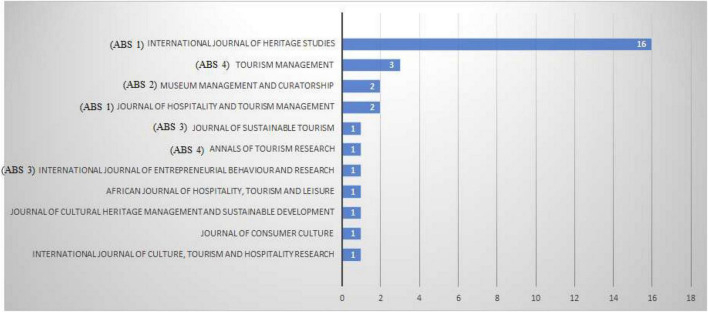
Number of articles per journal.

The first articles are published in 1990s (Evans-Pritchard, 1993; Shackley, 1997), but the inflection point occurred in 2015, with a growing number of publications from that date. The journals—and the respective number on the ABS ranking—where the articles have been published is shown in Figure 2.

**FIGURE 3:**
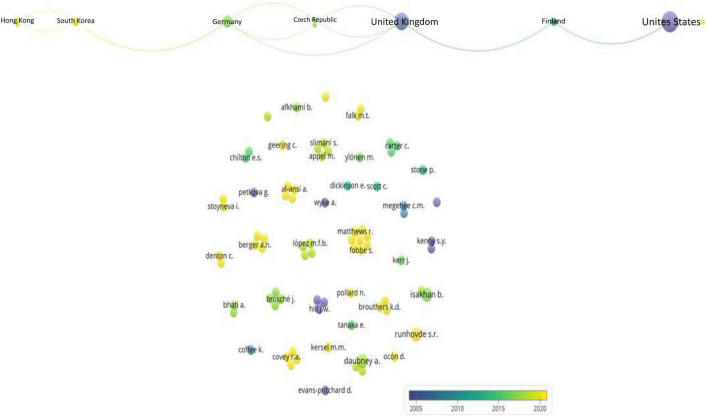
Network of authors and countries of affiliation.

The network of authors and countries of the universities of those who published in the field is illustrated in Figure 3. The United States and United Kingdom are the most prominent countries. Yet, the target of the study tends to be diversified for instance, Peru (Payntar et al., 2021), Norway (Runhovde, 2021), the Dead Sea (Kersel, 2021), Italy (Pollard, 2020), Iraq (Kathem, 2020), Spain (López et al., 2018), South Africa (Mofokeng, 2018), Bangkok Singapore (Bhati and Pearce, 2017), and Turkey (Tanaka, 2015).

## Cluster analyze

**FIGURE 4:**
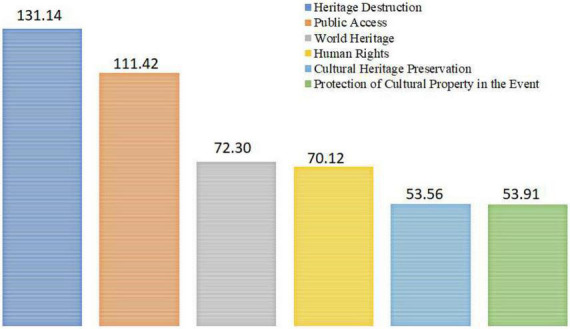
Text clustering. Only clusters with scores higher than 50 that represent concepts were considered. Score: shows the relevance value assigned to the cluster.

The text clustering returned six core clusters—representing concepts—shown in Figure 4, that is, heritage destruction, public access, world heritage, human rights, cultural heritage preservation, and protection of cultural property in the event. The figure also presents the score for each cluster.

### Heritage destruction

World War II led the world community to create diverse intergovernmental organizations dedicated to mapping critical locations and creating conventions and laws for their protection, as well as human rights, such as the United Nations and the United Nations Economic, Scientific and Cultural Organization (UNESCO). Different countries adopted their conventions and recommendations focused on humanitarian issues, such as the Universal Declaration of Human Rights, the Geneva Conventions, or the Universal Declaration of Human Rights, and the Genocide Convention. The destruction of cultural heritage or any work of art of any city, location or nation is regarded as an act of vandalism against the culture of our planet.

This cluster aggregated publication that deals with examples and situation where such world damage occurs. For instance, religious and political iconoclasm on the basis of the attack on various heritages sites in Iraq and Syria, that is, the mass looting of archeological zones, of ancient, buildings and statues, of religious and secular sites, museums, art galleries, and libraries (e.g., Brodie, 2015; Isakhan, 2015; Cunliffe et al., 2016; Isakhan and González Zarandona, 2018; Matthews et al., 2020).

Other studies discuss the concepts of protection and destruction to elaborate on how cultural objects should be dealt with (e.g., Baraldi et al., 2013; Tanaka, 2015). Yet, different stakeholders interpret protection and destruction in different ways, which causes issues when attempts to act in favor of cultural heritage, as happened in Turkey’s museum (Tanaka, 2015). Because of the unfortunate proliferation of local with heritage damages, Isakhan (2015) proposed a methodology for cataloguing heritage destruction in a database. This database prepared in Iraq can be extended to other nations. Aligned with heritage concerns, Bhati and Pearce (2017) developed and evaluated an observational approach to auditing the damage to tourist attractions in Asia: Bangkok and Singapore. The same process can be extended to other locations. Although academics tend to not focus on looting and plundering underwater sites, these sites deserve more attention in the future and the Bhati and Pearce’s (2017) tool should also be consider and adapted to such situations.

### Public access

Crowds in a heritage area can have a negative impact on cultural heritage, causing damage and property thefts (e.g., Stone, 2012; Grove et al., 2018; Al-Ansi et al., 2021). For instance, the floor can be damaged due to the flow of visitors, visitors can also leave trash, steal objects, or cause other damages (e.g., Evans-Pritchard, 1993; Egloff and Sayavongkhamedy, 2018; Grove et al., 2018). The public access and the respective damages caused can also be observed in looting and plundering underwater sites, such as shipwrecks (Grove et al., 2018). The theme of cultural heritage also includes the illegal trade in art and stolen pieces in the market (e.g., Hart and Chilton, 2015; Runhovde, 2021), which demands surveillance, knowledge and protection, and control systems.

Public access deserves more research in terms of flow of the tourists visiting heritage sites. Data mining and learning can make a huge contribution in predicting such flow and in reorganizing the flow of tourists. If tourists do not all pass through the same places and at the same time, the managers of these places will be contributing to their preservation. Therefore, the use of data mining and learning processes are a priority in research.

### World heritage

The concept of world heritage comes from UNESCO. This is a label that considers natural and cultural sites throughout the world. One important mission of such a label is to promote the protection of the places, destinations, or even transcends borders of nations. These sites are recognized as having worldwide relevance and represent examples of cultural or natural heritage. The situations of conflict and war represent threats to these world heritage sites (Geering, 2020). The concerns of mutilation, damage, and destruction of world heritage during the armed conflict is an issue that has deserved considerable attention (e.g., Zubrow, 2016; Brosché et al., 2017).

### Human rights

The right to education and the right of cultural and art belong to the Universal Declaration of Human Rights. Yet, war, vandalism, and destruction of heritage restricts such rights, leaving people and nations poorer, causing irreparable damage (e.g., Stone, 2012; Matthews et al., 2020). The priority of research in this cluster is to create replicas that can be stored and viewed from historical sites and monuments. This can be done with virtual and extended reality, as well as with the use of artificial intelligence algorithms. Thus, in case of war or natural disasters, humanity will be able to have a glimpse of what these ancestral spaces were like. Virtual reality can even contribute as reference information for the recovery of partially destroyed spaces or monuments.

### Protection of cultural property in the event

The Hague Convention for the Protection of Cultural Property in the Event of Armed Conflict of 1954 was created with the intention of protecting movable cultural property, such as works of art, museum collections, books, and archives (Geering, 2020). This cluster represents a small group of studies that examine damage and risk to cultural property sheltered in refuges, which were created due to specific events, as World War II (Pollard, 2020) or Cold War (Geering, 2020). Although sheltered the movable cultural properties have suffered careless military occupation, deliberate combatant damage, accidental and collateral damage, and looting (Pollard, 2020). Thus, the studies draw attention to movable cultural pieces and greater care to be taken with them in future dramatic events.

### Cultural heritage preservation

The damage due to the influx of visitors associated with looting and the walking and breathing of visitors have led cultural heritage mangers to use new technologies, such as virtual reality (VR), augmented reality (AR) and artificial intelligence (AI) and social media (e.g., Afkhami, 2017; Falk and Hagsten, 2020; Ocón, 2021). These technologies allow visitors to immerse themselves in cultural heritage sites and gain the experience of being there without actually being in the real world (VR). They also enhance the visitor experience, with complementary information or help to map, record, and organize information about cultural heritage and be guide (AI) (Loureiro et al., 2020; Loureiro, 2021). As Ocón (2021, p. 1) claims, “digitalization has reached cultural heritage” and “can help preserve its memories and lengthen its life.” The pandemic situation of COVID-19 forced citizens to stay at home and several cultural heritage places provide virtual visits (Loureiro, 2021), incrementing the use of such technologies.

## Research agenda

We develop future research lines focusing on the combination of research cluster with core actors in the cultural heritage context (e.g., Stamatoudi, 2011; Campelo et al., 2018). We recognize that technologies—as virtual (VR), augmented (AR) reality and artificial intelligence (AI)—has been gradually used in the tourism (e.g., Loureiro et al., 2020, 2021), but in the particular context of cultural heritage business and tourism management such technologies are still in an early stage of implementation. Thereby, we emphasize the encouragement of research on how technologies can benefit cultural heritage and contribute to preventing damage (see Table 1).

**TABLE 1:** Research suggestions.

Cluster	Actor	Research questions
Heritage destruction and Cultural heritage preservation	Tourism operators	° How can tourism operators entice tourists to visit cultural heritage sites and remind them not do damage the areas? ° How to create an international network with sites and artifacts in cooperation with tour operators to spread tourism and avoid crowds? ° How to create an international collaborative network of tour operators? Could they use GIS and other AI systems?
	Heritage managers	° How can heritage mangers create strategies to prevent heritage damage? How can they plan and implement them? ° How should organization managers implement AI systems in their organizations? ° How can AI systems assist managers of cultural heritage? How can such systems be implemented? ° How can a network of AI systems be able to connect different cultural heritages? ° How do heritage human workers need to be trained to operate with non-human AI systems and AI robots? ° How will human-AI robot interactions look?
	Local government	° How can local governments develop effective policies to protect heritage sites and artifacts? ° What about policies to protect cultural heritage datat and tourists that visit them? How can that big data be used in favor of world culture heritage preservation?
	Local communities	° How can local communities economically and culturally benefit with tourism in heritage sites? ° How to persuade local governments to direct funds to the local community?
	International trade	° How can art traders deal with damaged and looted art? ° How can multisensory virtual (where tourists use their five senses without actually being in the heritage site) tourism experiences be implemented into the context of cultural heritage for artifacts? ° How to develop business models with virtual representations of cultural heritage sites, natural, or artifacts?
Public access and human rights	Tourist and society	° How can the programs in high school and colleague be improved to sensitize students (citizens, tourists) to the preservation of cultural heritage? ° How can AI robots-virtual (e.g., holograms) and physical be designed (e.g., level of humanoid appearance, social capabilities) to achieve greater heritage tourist and society acceptance? ° How can multisensory virtual (where tourists use their five senses without actually being in the heritage site) tourism experiences be implemented into the context of cultural heritage for destinations? ° How can cultural heritage experiences be extended using AR technology? What will be different regarding sites, natural or artifacts heritage? ° How can virtual and/or augmented reality contribute to encourage heritage preservation?
World heritage and protection of cultural property in the event	International organizations	° How can international organizations (e.g., U.N.E.S.C.O., European Union) cooperate with cultural heritage sites and artifacts to protect cultural heritage against vandalism and war events? ° How can international organizations instill pro-cultural heritage preservation behaviors?

### Heritage destruction and cultural heritage preservation

Heritage destruction is the representative cluster in prior documents involving different actors, such as tourism operators, heritage managers, local governments, or local communities. Although it is very relevant to present case studies where heritage was destroyed and recommend that these situations should be avoided, future research should focus more on prevention. Although is quite relevant to present and describe case studies showing that cultural heritage has been destructed and recommendation to avoid such situation, future research should focus more on prevention. Another relevant aspect concerns the looting and plundering underwater sites, such as shipwrecks Academics so far have not paid due attention to these sites, so further studies are strongly recommended.

Thus, researchers can work together with different actors to contribute to create preservation strategies. Tourism operators have an important role in promoting heritage sites and artifacts but should also be more open to contribute to prevention and to avoid overcrowding. Technology can also contribute to mapping the heritage sites and the location of artifacts, helping to spread the tourists and visitors (e.g., GIS-Geographic Information System and other AI systems).

Heritage managers are core actors because the planification and organization of the heritage sites and artifacts depend on them. Although in cooperation with other stakeholders, they should lead the preservation and prevent damages in the heritage sites and artifacts. AI systems are capable of analyzing and processing large amounts of data (big data) giving managers tools to facilitate key decisions. They collect, aggregate, analyze, compare, and interact, being even able to take some decisions and learn with previous situations and interactions conducted. Thus, researchers have the opportunity to investigate how AI systems can be implemented and how to create integrate networks of AI toward a more efficiency management of different heritage sites in the world. Managers should also be aware that in interactions between humans and non-humans (AI agents or robots), human workers need training. In this new work system, where humans interact with non-humans, issues such as work tasks, ethics and politics will arise.

Local governments need to operate in tandem with heritage managers to develop policies to organize and protect the cultural heritage sites and artifacts. Heritage managers and international traders benefit from cooperating with each other through the exchange of data. VR and AR have been used to enhance the experience of visiting museums and heritage sites and can also be a support for heritage artifacts. For instance, instead of handling an old book, tourists and potential buyers will be able to experience this through VR. This virtual experience can prevent damage to the artifact due to breathing and handling. This way, more research is needed to understand how tourists and potential buyers can experience cultural heritage virtually. New business models will be developed using VR, AR, and AI systems and cultural heritage.

### Public access and human rights

Public access and human rights clusters are more associated with tourists and society. Future studies should be more concerned with education for the preservation of cultural heritage. Basic and higher education levels should be more concerned with incorporating issues of destruction and preservation of cultural heritage into their tourism courses to develop a global awareness of cultural heritage.

Researchers should be more open to develop research on the acceptance of AI robots to support tourists in visiting cultural heritage sites and artifacts. AI robots-virtual (e.g., holograms) and physical (e.g., level of humanoid appearance, social capabilities) can perform an important role in in serving, guiding, and informing visitors about the history of the sites and managing the flow of visitants.

The multisensory virtual tourism experiences are also a theme to be explored, since virtual sites, instead of real ones, can avoid overcrowding and consequently contribute to preserve heritage sites. The combination of virtual and augmented reality can even make experiences more exciting and vivid. We recommend analyzing several concepts, such as tourist’s emotions, subjective well-being, authenticity perception, inspiration, self-connection, or cultural expertise. Hence, more studies employing mixed approach and quantitative data treatment are suggested.

### World heritage and protection of cultural property in the event

International organizations have performed a fundamental work in drawing worldwide attention to the relevance to the culture and citizen identity the preservation of nature, destinations, sites, and artifacts. For instance, UNESCO encourages countries to sign the World Heritage Convention of 16 of November of 1972 and ratified on 1975, to create plans for its protection, and to provide emergency assistance for situations of immediate danger. Yet, heritages sites are vandalized and destroyed for religious, political, and war reasons. So, what else should be done? How can these organizations act in such dramatic situation? How can they promote education and knowledge to gain more members for the cause?

Academics should conduct research on tourists and citizens pro-cultural heritage preservation. In another words, academics need to investigate what can drive—cognitively, emotionally and relationally—tourists and citizens in different cultural context and counties to preserve cultural heritage.

The war events cause by humans and or those due to natural causes should not be neglected. International organization should operate near by the heritage managers and local governments to plan how to reduce damage when such events occur. Academics should develop more accurate forecasting models to predict natural disasters, which can give managers time to rescue cultural heritage.

## Conclusion

This study uses text mining to give an overview of the network of authors and counties and extract the main clusters of the themes analyzed in prior studies to create suggestions for future research. Although the first Scopus indexed article—in the field of business and tourism management—goes back to the 1990s, this theme of cultural heritage and looting has not received due attention from researchers. This study can be a call for more research in the field of tourism management.

This study contributes by highlighting the publications—books and above all the articles—journals, authors, countries, and clusters that have been in the heart of the discussion of the topic. We also contribute to academics and managers by presenting the cluster analysis and the research suggestions.

Academics can benefit from this study by having a comprehensive review of the literature on cultural heritage and looting, the network of researchers and the cluster themes. The suggestions for future research and the research questions offer academics the possibility to strategically organize themselves and prepare the next steps of their future research (see Table 1). Thus, we offer (1) the clusters emerging on cultural heritage and looting and (2) new avenues for future research that give high value for the academic development in cultural heritage and looting. The six clusters from prior research adds to academia by clearly expose what have been discussed on cultural heritage and looting.

This study unveils to business and tourism managers what has been discussed on the topic of cultural heritage and looting. They can use such information to alert themselves about the risks and benefits associated with cultural heritage sites, artifacts, and destinations in terms of destruction, preservation, overcrowding and human rights. This knowledge can contribute to help business and tourism managers to strategically manage their business, tourist sites, artifacts, and destinations. This study contributes to tourism management because summarize the relevant topics that has been discussed in literature: heritage destruction, public access, world heritage, human rights, protection of cultural property in the event, and cultural heritage preservation. Heritage destruction deals with religious and political iconoclasm that contribute to the destruction of monuments and heritage sites. Public access is dedicated to the flow of tourists in heritage sites. World heritage gives examples on sites recognized as relevant worldwide. The right to education of cultural and art belongs to the Universal Declaration of Human Rights and the cluster human rights is dedicated to those rights. Protection of cultural property in the event is dedicated to damage and risk to cultural property sheltered in refuges and finally immersive and extended technologies can be used to give visitors different perspectives in cultural heritage. Therefore, the current paper highlights others that can be read by managers depending on the topic that is more relevant for them and their institutions.

Regarding limitations, we focused our study on business and tourism management, but eventually we can find other interesting documents in other fields. Although WoS and Scopus are two well-reputed and known databases aggregating different publishers (e.g., Wiley, Emerald, Elsevier, Taylor and Francis, Sage), future studies can consider other databases to search for related documents on the topic. Lastly, this research centers on the qualitative text-mining review process. For future research, integrating a quantitative process for exploring the role of core clusters identified in this research is suggested.

## Data availability statement

The raw data supporting the conclusions of this article will be made available by the authors, without undue reservation.

## Author contributions

All the authors contributed to conceptualization, formal analysis, investigation, methodology, writing and editing the original draft, and approved the submitted version.
